# Febuxostat Alleviates Allergic Rhinitis by Inhibiting Inflammation and Monocyte Adhesion in Human Nasal Epithelial Cells via Regulating KLF6

**DOI:** 10.1155/2022/9092311

**Published:** 2022-09-08

**Authors:** Yuting Yao, Ran Wei, Hui Jiang

**Affiliations:** Otorhinolaryngology, Dongfang Hospital Beijing University of Chinese Medicine, No. 6 Fangxingyuan, Fengtai District, Beijing 100078, China

## Abstract

**Introduction:**

Febuxostat is a novel inhibitor of xanthine oxidase that suppresses cell adhesion molecules-mediated (CAMs) inflammation by activating KLF6. In this study, we explored the therapeutic function and potential mechanisms of febuxostat against allergic rhinitis (AR).

**Methods:**

We investigated the role of febuxostat through *in vitro* cell and *in vivo* animal experiments. Human nasal epithelial cells (hNECs) were cultured with histamine as an *in vitro* model. To establish the AR animal model, rats were exposed to ovalbumin. Rats were randomly grouped into control, model, 7.5 mg/kg febuxostat, and 15 mg/kg febuxostat groups.

**Results:**

In the *in vitro* study, we found significantly increased release of lactate dehydrogenase, elevated production of inflammatory factors and chemokines, and upregulated CAMs in histamine-treated hNECs. However, these results were significantly reversed for the 10 and 20 *μ*M febuxostat treatments. The enhanced adhesion between hNECs and monocytes induced by histamine was dramatically repressed by febuxostat. In the *vivo* experiments, we observed that febuxostat ameliorated the increased sneezing times, the number of nose scratching episodes, and elevated HE pathological scores as well as alleviated the inflammation in nasal mucous tissues of AR mice. We found that KLF6, which was downregulated in histamine-treated hNECs, was significantly upregulated by febuxostat. The inhibitory effects of febuxostat on the expression levels of CAMs and adhesion between histamine-treated hNECs and monocytes were significantly abolished by the knockdown of *KLF6*.

**Conclusion:**

Febuxostat alleviates AR by inhibiting inflammation and monocyte adhesion in human nasal epithelial cells through the regulation of KLF6.

## 1. Introduction

Allergic rhinitis (AR) is a common rhinitis with clinical symptoms such as itchy nose, sneezing, and runny nose, which cause significant inconvenience in the normal lives of AR patients [1]. At present, approximately 30% of the global population is affected by AR and the morbidity rate in America is 5%-22%. The annual cost for AR treatment in America is approximately 7 billion dollars [2]. In addition, with economic development, acceleration of industrialization, and lifestyle changes in China, the morbidity rate of AR has increased. It has been reported that approximately 30 million patients are diagnosed with AR in China every year [3, 4]. Therefore, AR has triggered significant public health problems worldwide.

After AR patients are stimulated with allergens, immunoglobulin E (Ig E) is excessively produced in the nasal mucosa and binds with the receptor located on mast cells, which further induces degranulation in mast cells and the release of multiple inflammatory mediators such as histamine and leukotrienes. Consequently, the development of AR is induced under the regulation of multiple inflammatory cells and inflammatory factors [5]. The pathological mechanism of AR is complicated. Currently, the theory of imbalance between type 1 helper T (Th1) and type 2 helper T (Th2) cells is widely accepted [6]. The relationship between the pathogenesis of AR and cytokines has been extensively investigated such as interleukin 4 (IL-4) and interleukin 9 (IL-9) released by Th2 cells [7, 8], interleukin 5 (IL-5) produced by CD4^+^ T cells and mast cells [9], interleukin 12 (IL-12) released by antigen-presenting cells (APCs) [10], and tumor necrosis factor-alpha (TNF-*α*) secreted by macrophages [11]. Additionally, the disorder of the cytokine network is the molecular basis for developing AR [12]. For investigating the pathological mechanism of AR, the function of cell adhesion molecules (CAMs) released by Th cells in the development of AR has received considerable attention [13]. The infiltration and adhesion of inflammatory cells in local or systemic inflammatory tissues are keys to the progression of inflammation. In clinical investigations, a significantly higher number of neutrophils, eosinophils (EOS), and lymphocytes are observed in AR patients [14]. The involvement of inflammatory cells in immunologic processes depends on the regulation of CAMs [15]. Recently, KLF6, which is a transcriptional factor, was reported to suppress the expression of CAMs to mediate the inflammatory reaction [16]. Therefore, KLF6 might be an important target for the treatment of clinical AR due to its function in regulating CAMs-mediated inflammation.

Febuxostat is a novel inhibitor of xanthine oxidase and was developed for treating hyperuricemia by reducing the production of uric acid [17]. Animal experiments revealed that febuxostat showed a promising protective property in renal ischemia-reperfusion injury [18], diabetic nephropathy (DN) [19], and myocardial ischemia-reperfusion injury [20] by inhibiting oxidative stress. Recently, febuxostat was reported to suppress adhesion between monocytes and endothelial cells by regulating the expression level of CAMs, which is associated with the activation of KLF6 [21]. In this study, we explored the inhibitory function of febuxostat on inflammation to determine the therapeutic function of febuxostat on AR.

## 2. Materials and Methods

### 2.1. Cells and Treatments

Human nasal epithelial cells (hNECs) and U937 cells were obtained from ATCC (ATCC, California, USA) and cultured in a DMEM medium containing 10% FBS under the condition of 37°C and 5% CO_2_.

### 2.2. The MTT Assay

After different treatment strategies, cells were added with 0.25 mg/ml MTT (Sigma, Missouri, USA) at 37°C for 3 h, followed by removing the medium and adding the dimethyl sulfoxide for the production of blue formazan. Then, the microplate reader (Mindray, Shenzhen, China) was used to measure the absorbance at 630 nm.

### 2.3. The Lactate Dehydrogenase (LDH) Release Assay

In brief, treated hNECs were seeded in the 96-well plate to be incubated for 24 hours. Subsequently, the collected supernatants were added to the CytoTox 96 Reagent (Promega, Wisconsin, USA), followed by incubating it with the stop solution. Lastly, the percentage of LDH released was calculated by the following equation: LDH releases (%) = (experimental LDH release spontaneous LDH release)/maximum LDH release.

### 2.4. Real-Time qPCR

The trizol reagents were utilized to extract the cellular total RNA from treated hNECs and the concentration of RNA was measured by detecting the optical density at 260 nm, followed by being transcribed into cDNA by utilizing the PrimeScript RT Master Mix Kit (Takara, Tokyo, Japan). The Sybr Premix Ex Taq Kit (Takara, Tokyo, Japan) was used to perform the RT-qPCR in the present study. The 2^−ΔΔCt^ method was utilized to calculate the expression of genes after normalization to Gapdh. The primers used in the qRT-PCR analysis are as follows:

  Gapdh primer F: 5′- GCACCGTCAAGGCTGAGAAC -3′
  Gapdh primer R: 5′- TGGTGAAGACGCCAGTGGA -3′
  IL-6 primer F: 5′-TCCAGAACAGATTTGAGAGTAGTG-3′
  IL-6 primer R: 5′-GCATTTGTGGTTGGGTCAGG-3′
  TNF-*α*primer F: 5′-CCTGTGAGGAGGACGAAC-3′
  TNF-*α* primer R: 5′-CCTGTGAGGAGGACGAAC-3′
  IL-12 primer F: 5′-TTCTTATCGATATGGGTCAC CAGCAGTTGGTCAT-3′
  IL-12 primer R: 5′-TTTTTATCGATGGAAGCATTCAGATAGCTCATCA-3′

### 2.5. ELISA Assay

The secretion of cytokines was determined by the ELISA assay (Elabscience, Wuhan, China). In brief, 5 gradient concentrations of the standard were obtained and added to the 96-well plates, along with the supernatant collected from the culture medium of each group. The samples and the standards were incubated in the wells for half an hour at 37°C, followed by removing the medium and washing it 3 times. Then, conjugate reagents were added into each well to be incubated for half an hour at 37°C. After adding 3,3′,5,5′-Tetramethyl benzidine (TMB) solution it was incubated again for 15 min, and the stop reagent was introduced to end the reaction. Lastly, the microplate reader (Bio Tek, Vermont, USA) was used for the detection of absorbance at 450 nm.

### 2.6. Western Blotting Assay

Following the extraction of total proteins from the treated hNECs using the lysis buffer, a BCA kit was used to quantify the isolated proteins and approximately 40 *μ*g proteins were loaded and separated by 12% SDS-PAGE and transferred to the PVDF membrane (Millipore, Massachusetts, USA). The membrane was then incubated with 5% BSA followed by incubation with the primary antibody against VCAM-1 (1 : 1000, Affinity Biosciences, Melbourne, Australian), E-Selectin (1 : 1000, Affinity Biosciences, Melbourne, Australian), KLF6 (1 : 1000, Affinity Biosciences, Melbourne, Australian), and Gapdh (1 : 1000, Affinity Biosciences, Melbourne, Australian). Subsequently, the membrane was incubated with the secondary antibody (1 : 2000, Affinity Biosciences, Melbourne, Australia). Lastly, the bands were visualized by ECL solution, and the relative expression level of target proteins was quantified by the Image J software.

### 2.7. Calcein Acetoxymethyl Ester (Calcein Am) Staining

The attachment of U937 monocytes to hNECs was measured by the calcein-AM staining assay. After being stained with 1 *μ*M calcein-AM in the dark for 30 min, 5 × 10^5^ U937 monocytes were added to approximately 1 × 10^5^ hNECs for 2 hours, followed by removing the unbounded cells and visualizing the attached cells under a fluorescent microscope (Olympus, Tokyo, Japan).

### 2.8. AR Modeling and Grouping

24 male C57BL/6 mice were obtained from Shanghai SLAC Laboratory Animal Co.,Ltd (Shanghai, China) and 18 mice were used to establish the AR model, with the remaining 6 mice as the control group. For the establishment of the AR model, mice were injected with ovalbumin (OVA) and aluminium hydroxide intraperitoneally, followed by stimulation on days 21, 28, 35, and 39 postinjection using OVA nasal drops. Animals in the control group were administered with normal saline and nasal drops with ultrapure water on days 21, 28, 35, and 39 postinjection.They were then divided into following four groups: control, model, 7.5 mg/kg febuxostat, and 15 mg/kg febuxostat. Animals in the control (normal mice) and model group (AR mice) were dosed with normal saline for 21 days. AR mice in the 7.5 mg/kg febuxostat group and in the15 mg/kg febuxostat group were administered orally with 7.5 mg/kg/day febuxostat and 15 mg/kg/day febuxostat for 21 days, respectively [22].

### 2.9. Behavioral Experiments

The symptom of AR was evaluated by sneezing times and the number of nose scratchesin 15 mins, which were recorded for the statistical analysis.

### 2.10. Hematoxylin and Eosin (HE) Staining

After collecting the nasal mucous tissues from each mouse, tissues were dehydrated with different concentrations of ethanol solution and ethanol and xylene until the tissues looked transparent, followed by embedding, sectioning and staining them with H&E. Lastly, the images were taken using the inverted microscope (Leica, Wetzlar, Germany). The degree of pathology was rated as follows: 0–5 points: 0: no lesion; 1: very mild lesion; 2: mild lesion; 3: moderate lesions; 4: severe lesions; and 5: extremely severe lesions.

### 2.11. Transfection

The specially designed siRNA against KLF6 was synthesized by Nanjing Genscript (Nanjing, China), the sequences of which were 5′ CACACAGGAGAAAAGCCUUACAGAU-3′. In brief, cells were seeded in 6-well plates, followed by the addition of 40 nM siRNAs and lipofectamine 2000 (Sigma-Aldrich, Missouri, USA), following which the transfecting efficacy was evaluated using the Western blotting assay.

### 2.12. Statistical Analysis

Data were analyzed using the Graphpad software and were presented as the mean ± SD. Students't test was used to compare two independent data and the data among groups were compared using the one-way ANOVA method, while *p* < 0.05 was taken as the significant difference.

## 3. Results

### 3.1. Screening the Optimized Concentration of Febuxostat

**Figure 1:**
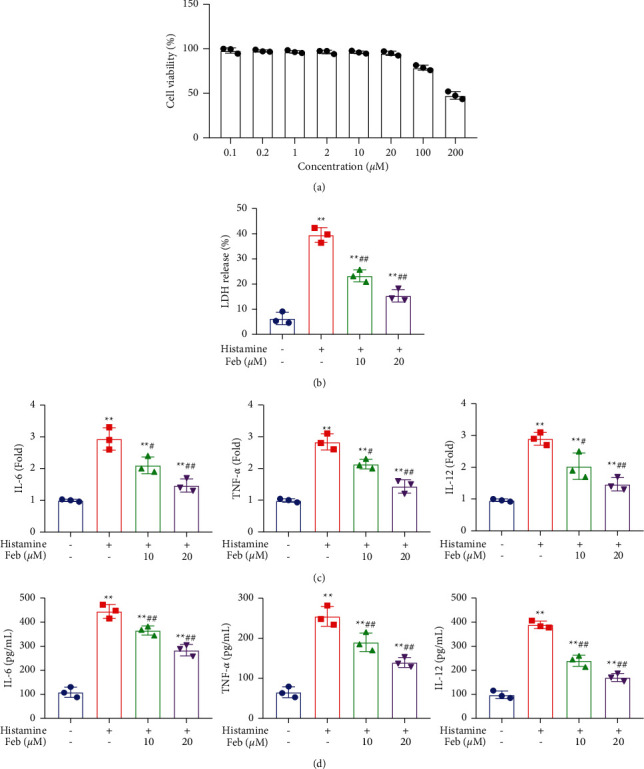
The cell injury and inflammatory state in histamine-treated hNECs were alleviated by Febuxostat. (a) The cell viability was detected by utilizing the MTT assay, (b) LDH release was measured, (c) the expression level of IL-6, TNF-*α*, and IL-12 was determined by RT-qPCR assay, and (d) the release of IL-6, TNF-*α*, and IL-12 was measured by the ELISA assay (*p* < 0.01).

To obtain an optimized concentration of febuxostat, hNECs were incubated at different concentrations (0.1, 0.2, 1, 2, 10, 20, 100, and 200 *μ*M) for 24 hours, followed by an evaluation of cell viability using the CCK-8 assay. Cell viability (Figure 1(a)) was slightly changed under concentrations ranging from 0.1 to 20 *μ*M. When the concentration was higher than 100 *μ*M, cell viability dramatically declined. In subsequent experiments, 10 and 20 *μ*M were used for incubation.

### 3.2. Febuxostat Alleviated the Inflammatory State in Histamine-Treated hNECs

hNECs were treated with 0.1 *μ*M histamine [23] in the absence or presence of 10 and 20 *μ*M febuxostat, respectively. hNECs cells without treatment were utilized as the negative control. Compared to the control, we found that the LDH release (Figure 1(b)) was significantly elevated from 5% to 40.1% by the stimulation of histamine, which was dramatically suppressed by 23.9% and 15.0% in the 10 and 20 *μ*M febuxostat groups, respectively. The upregulated IL-6, TNF-*α*, and IL-12 (Figure 1(c)) in histamine-stimulated hNECs were dramatically downregulated by the treatment with 10 and 20 *μ*M febuxostat. Compared to the control, the release of TNF-*α* (Figure 1(d)) was greatly promoted from 72.9 pg/mL to 254.6 pg/mL in the histamine group and significantly declined to 186.8 pg/mL and 145.1 pg/mL in the 10 and 20 *μ*M febuxostat group, respectively. Concentrations of IL-6 in the control, histamine, 10, and 20 *μ*M febuxostat groups were 105.2, 445.1, 368.3, and 296.8 pg/mL, respectively. Finally, the production of IL-12 was greatly elevated from 99.4 pg/mL to 402.2 pg/mL in the histamine group, and significantly decreased to 245.7 pg/mL and 176.6 pg/mL in the 10 and 20 *μ*M febuxostat groups (*p* < 0.01), respectively. These results imply that cell injury and the inflammatory state in hNECs induced by histamine were significantly alleviated by febuxostat.

### 3.3. Febuxostat Suppressed the Expression Level of Chemokines and Adhesion Molecules in Histamine-Treated hNECs

**Figure 2:**
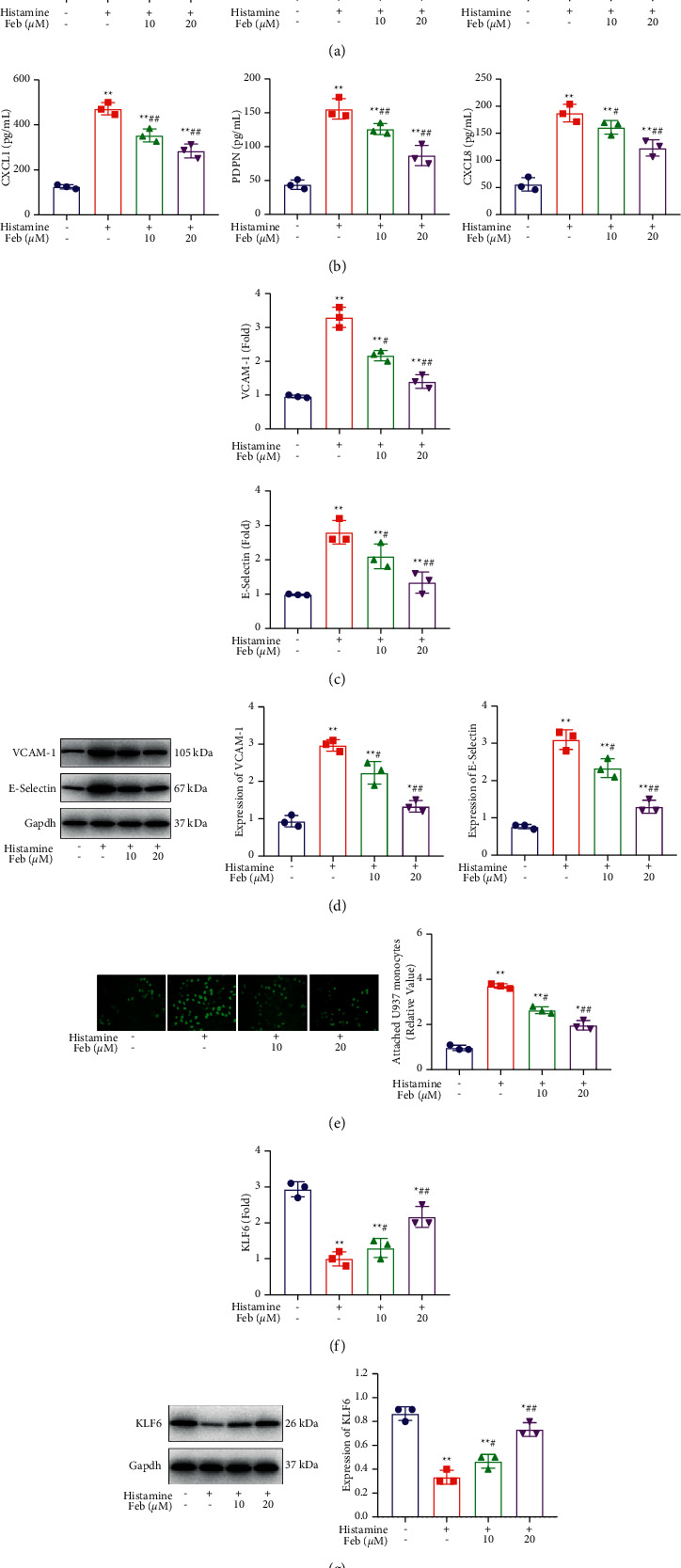
The expression level of chemokines, adhesion molecules, and KLF6 expression in histamine-treated hNECs were repressed by febuxostat. (a). The expression level of chemokines was measured by the RT-qPCR assay, (b) the production of chemokines was evaluated by the ELISA assay, (c) the expression level of CAMs was checked by the RT-qPCR assay, (d) the expression level of CAMs was measured by the Western blotting assay (*p* < 0.05 and *p* < 0.01), (e) the adhesion between hNECs and U937 monocytes was inhibited by febuxostat. The attached U937 monocytes were detected using the calcein-AM staining assay (*p* < 0.05 and *p* < 0.01), (f) the expression level of KLF6 was evaluated by the RT-qPCR assay, and (g) the expression level of KLF6 was determined by the Western blotting assay (*p* < 0.05 and *p* < 0.01). Immunofluorescence magnification was 10×.

We explored the impact of febuxostat on the expression level of chemokines and adhesion molecules to explore potential mechanisms. CXCL-1, PDPN, and CXCL8 (Figure 2(a)) were dramatically upregulated by histamine, which was significantly reversed by 10 and 20 *μ*M febuxostat. Compared with the control, the secretion of CXCL-1 (Figure 2(b)) significantly increased from 125.3 pg/mL to 488.5 pg/mL in the histamine group, which was significantly decreased to 389.8 pg/mL and 323.6 pg/mL in the 10 and 20 *μ*M febuxostat group, respectively. Concentrations of PDPN in the control, histamine, 10, and 20 *μ*M febuxostat groups were 49.6, 152.4, 129.3, and 97.0 pg/mL, respectively. Finally, the release of CXCL8 was dramatically promoted from 63.2 pg/mL to 183.7 pg/mL in the histamine group and was dramatically suppressed to 157.5 pg/mL and 126.1 pg/mL in the 10 and 20 *μ*M febuxostat groups, respectively. VCAM-1 and E-Selectin (Figures 2(c) and 2(d)) were greatly upregulated in the histamine group, which was significantly downregulated by 10 and 20 *μ*M of febuxostat (*p* < 0.05 and *p* < 0.01), respectively.

### 3.4. Febuxostat Inhibited Adhesion between hNECs and U937 Monocytes

To confirm the effect of febuxostat on adhesion between hNECs and inflammatory cells, a calcein-AM staining assay was performed. The attached U937 monocytes (Figure 2(e)) were dramatically promoted in the histamine group but 10 and 20 *μ*M febuxostat significantly and dose-dependently suppressed these (*p* < 0.05 and *p* < 0.01, respectively). This indicated a promising inhibitory effect of febuxostat on the attachment between hNECs and U937 monocytes.

### 3.5. Febuxostat Elevated the Expression Level of KLF6 in Histamine-Treated hNECs

We explored the effects of febuxostat on KLF6 and found that compared to the control, KLF6 (Figures 2(f) and 2(g)) was greatly downregulated in histamine-treated hNECs. Additionally, this was significantly reversed by 10 and 20 *μ*M febuxostat (*p* < 0.05 and *p* < 0.01, respectively), which indicated that KLF6 might be involved in the therapeutic function of febuxostat.

### 3.6. Febuxostat Ameliorated the Pathological Symptoms of AR Mice

**Figure 3:**
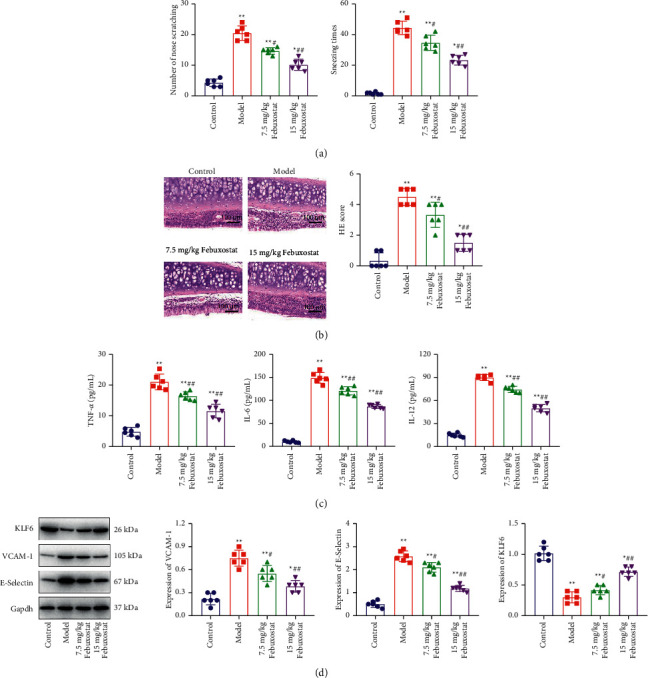
The AR pathological symptom was alleviated by febuxostat. (a) The sneezing times and the number of nose scratching were recorded, (b) the pathological state in nasal mucosa tissues was determined by HE staining, (c) the secretion of TNF-*α*, IL-6, and IL-12 in nasal mucosa tissues was measured by the ELISA assay (*p* < 0.05 and *p* < 0.01), and (d) the expression level of CAMs and KLF6 were measured by the Western blotting assay (*p* < 0.05 and *p* < 0.01).

To verify the therapeutic effect of febuxostat on AR, AR mice were treated with 7.5 mg/kg/day and 15 mg/kg/day for 21 consecutive days and evaluated for pathological changes. We found that compared to the control group, sneezing times and the number of nose scratches (Figure 3(a)) were significantly elevated in the model group but were dramatically decreased in the 7.5 mg/kg and 15 mg/kg febuxostat groups. The results of HE staining on nasal mucous tissues (Figure 3(b)) indicated that compared to the control group, significant infiltration of inflammatory cells and increased HE scores were observed in the model group but these results were dramatically abolished by 7.5 mg/kg and 15 mg/kg of febuxostat. Additionally, elevated production of TNF-*α*, IL-6, and IL-12 in nasal mucous tissues (Figure 3(c)) in AR mice was greatly suppressed by 7.5 mg/kg and 15 mg/kg of febuxostat (*p* < 0.05 and *p* < 0.01, respectively). Results of the expression of CAMs (VCAM-1 and E-selectin) and KLF6 (Figure 3(d)) were consistent with detection results of inflammatory factors (TNF-*α*, IL-6, and IL-12). These results collectively revealed that pathological changes in AR mice were significantly ameliorated by febuxostat.

### 3.7. Febuxostat Inhibited Adhesion between hNECs and U937 Monocytes by Activating KLF6

**Figure 4:**
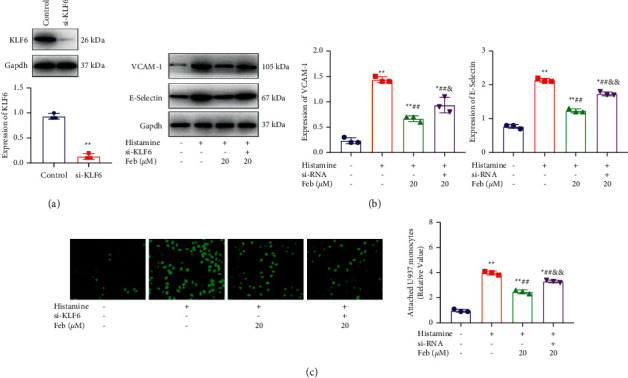
The inhibitory effect of febuxostat on the adhesion between hNECs and U937 monocytes. (a) The knockdown efficacy on KLF6 was evaluated by the Western blotting assay, (b) the expression level of CAMs was determined by theWestern blotting assay, and (c) the adhesion between hNECs and U937 monocytes was inhibited by febuxostat. The attached U937 monocytes were detected using the calcein-AM staining assay (*p* < 0.05 and *p* < 0.01). Immunofluorescence magnification was 10×.

To verify the febuxostat mechanism, hNECs were transfected with siRNA targeting KLF6 to establish KLF6-knockdown hNECs. The knockdown efficacy (Figure 4(a)) was identified using a Western blotting assay (*p* < 0.01). Subsequently, hNECs were introduced with febuxostat in the presence or absence of siRNA-KLF6. The elevated expression level of VCAM-1 and E-selectin (Figure 4(b)) in histamine-treated hNECs was significantly repressed by febuxostat, which was significantly abolished by the knockdown of KLF6. The increased attachment of U937 monocytes in histamine-treated hNECs was dramatically inhibited by febuxostat (Figure 4(c)) but this was greatly reversed by the knockdown of KLF6 (*p* < 0.05 and *p* < 0.01, respectively). These results indicate that the inhibitory function of febuxostat on adhesion between hNECs and U937 monocytes may be mediated by the activation of KLF6.

## 4. Discussion and Conclusion

CAMs are a group of molecules that mediate the interaction among cells or between cells and a matrix, which are mainly distributed in the extracellular matrix (ECM) and exert regulatory functions by binding to specific receptors [24]. CAMs are involved in multiple types of pathological and physiological processes including the recognition of cell types, activation of cellular signal pathways, maintenance of the cellular structure, wound healing, coagulation, and tumor metastasis [25, 26]. CAMs are mainly divided into four categories as follows: the selectin family, integrin family, immunoglobulin superfamily, and cadherin family [27]. During the pathological progression of AR, CAMs bind with lymphocyte function-associated antigens to induce adhesion between T-cells and target cells such as antigen-presenting cells (APCs), epithelial cells, and endothelial cells, which further mediate the selective accumulation and adhesion of inflammatory cells including eosinophils (EOS). The accumulation and adhesion of inflammatory cells are closely related to the development of AR [28, 29]. In this study, *in vitro* results indicated that excessive production of inflammatory factors and elevated expression levels of CAMs were observed in histamine-treated hNECs, which was similar to a previous report [30]. After the treatment with febuxostat, the secretion of inflammatory factors and expression of CAMs in histamine-treated hNECs were dramatically reversed, which indicated a promising anti-inflammatory effect of febuxostat against histamine-induced inflammation.

Compared to healthy subjects, in AR patients, the expression of adhesion molecules was significantly and highly expressed in vascular endothelial cells, gland cells, submucosal lymphocytes, and EOS cells [31]. Under the stimulation of inflammation, CAMs were further upregulated by the induction of elevated and released TNF-*α* and IL-1 in EOS and mast cells [32, 33]. Dissociative CAMs, such as VCAMs, were significantly upregulated in the serum when the intranasal allergen was activated [34]. The important role of CAMs in the pathogenesis of AR is currently receiving considerable attention [35]. Functional experiments in this study revealed that the adhesion between histamine-treated hNECs and monocytes was significantly repressed by febuxostat, and this verified the inhibitory effect of febuxostat on CAMs. *In vivo* experiments indicated that AR pathological symptoms were significantly ameliorated by febuxostat. This was also accompanied by the alleviation of the inflammatory state in nasal mucous tissues in AR mice, which indicated that the therapeutic effect of febuxostat on AR was mediated by its anti-inflammatory properties.

Krüppel-like factor (KLF)6 is a nuclear transcription factor commonly located in mammals and is also called zinc finger factor 9(Zf9) or core promoter element binding protein. The carboxyl-terminal of KLF6 is composed of 3 sequential C_2_H_2_ type zinc finger structures, which regulate the expression level of downstream genes by specifically targeting and binding the CACCC and GC cassette located in the promoter [36, 37]. KLF6 is involved in multiple types of cellular progression, such as cell development, growth, signaling transition, proliferation, differentiation, and apoptosis, and the deficiency of KLF is closely associated with the development of malignant tumors [38, 39]. Recently, KLF6 was reported to regulate the expression of CAMs and mediate adhesion between endothelial cells and monocytes [21, 40]. In this study, we found that KLF6 was dramatically downregulated in histamine-treated hNECs but elevated by febuxostat, which is consistent with previous reports [21]. Further, we found that the inhibitory effects of febuxostat on the expression of CAMs and adhesion between histamine-treated hNECs and monocytes were significantly abolished by the knockdown of KLF6, which indicated that febuxostat exerted an anti-inflammatory effect by activating KLF6. In future studies, the direct interaction between febuxostat and KLF6 will be investigated to determine molecular regulatory mechanisms underlying the effects of febuxostat on monocyte adhesion and inflammation. Collectively, our data indicate that febuxostat alleviates AR by inhibiting inflammation and monocyte adhesion in human nasal epithelial cells through the regulation of KLF6.

## Data Availability

The data used to support the findings of this study are included within the article.
